# Age-related DNA methylation changes are tissue-specific with *ELOVL2* promoter methylation as exception

**DOI:** 10.1186/s13072-018-0191-3

**Published:** 2018-05-30

**Authors:** Roderick C. Slieker, Caroline L. Relton, Tom R. Gaunt, P. Eline Slagboom, Bastiaan T. Heijmans

**Affiliations:** 1 Molecular Epidemiology, Department of Biomedical Data Sciences, Leiden University Medical Center, Einthovenweg 20, 2333 ZC Leiden, The Netherlands; 2 MRC Integrative Epidemiology Unit, School of Social and Community Medicine, University of Bristol, Bristol, BS8 2BN UK

**Keywords:** DNA methylation, 450 k, Tissue-specific, Ageing

## Abstract

**Background:**

The well-established association of chronological age with changes in DNA methylation is primarily founded on the analysis of large sets of blood samples, while conclusions regarding tissue-specificity are typically based on small number of samples, tissues and CpGs. Here, we systematically investigate the tissue-specific character of age-related DNA methylation changes at the level of the CpG, functional genomic region and nearest gene in a large dataset.

**Results:**

We assembled a compendium of public data, encompassing genome-wide DNA methylation data (Illumina 450k array) on 8092 samples from 16 different tissues, including 7 tissues with moderate to high sample numbers (Dataset size range 96–1202, *N*_total_ = 2858). In the 7 tissues (brain, buccal, liver, kidney, subcutaneous fat, monocytes and T-helper cells), we identified 7850 differentially methylated positions that gained (gain-aDMPs; cut-offs: *P*_bonf_ ≤ 0.05, effect size ≥ 2%/10 years) and 4,287 that lost DNA methylation with age (loss-aDMPs), 92% of which had not previously been reported for whole blood. The majority of all aDMPs identified occurred in one tissue only (gain-aDMPs: 85.2%; loss-aDMPs: 97.4%), an effect independent of statistical power. This striking tissue-specificity extended to both the functional genomic regions (defined by chromatin state segmentation) and the nearest gene. However, aDMPs did accumulate in regions with the same functional annotation across tissues, namely polycomb-repressed CpG islands for gain-aDMPs and regions marked by active histone modifications for loss-aDMPs.

**Conclusion:**

Our analysis shows that age-related DNA methylation changes are highly tissue-specific. These results may guide the development of improved tissue-specific markers of chronological and, perhaps, biological age.

**Electronic supplementary material:**

The online version of this article (10.1186/s13072-018-0191-3) contains supplementary material, which is available to authorized users.

## Background

The association between DNA methylation and age in humans is well established for whole blood [1–10], and also in adipose tissue, brain and mesenchymal stem cells, loci have been found where DNA methylation changes with age [11–13]. A prime example is the CpGs near the *ELOVL2* gene that exhibit consistent age-related changes in blood, hMSCs [13] and teeth [14] and other tissues [15, 16], an association that even extends to tissue from another species, namely the mouse [17]. The strength of the associations has led to the development of multiple predictors that can accurately estimate chronological age from methylation levels at a limited set of CpG sites [18–20]. While most predictors are trained on whole-blood DNA methylation data, one age predictor works independent of tissue type [18–20]. Intuitively, the high precision of the tissue-independent age predictor may rely on combining the cumulative information of CpGs whose DNA methylation level changes with age in multiple tissues simultaneously [14]. However, current views of the extent of tissue-specificity versus tissue-shared age-related DNA methylation are based on relatively small-scale studies with repect to the number of samples, tissues and/or CpG sites (Table 1). The three previous human studies on tissue-specificity included between 4 and 92 samples per tissue (and 656 whole-blood samples) interrogating 1413, 26,486 and 429,789 CpG sites [13, 21, 22]. Although two of these studies concluded that age-related DNA methylation are tissue-specific [13, 21], the third reported that age-related changes were both shared across tissues and tissue-specific [22]. However, small numbers of tissues, samples and CpGs are biased towards finding tissue-specificity. Hence, conclusive evidence whether age-related changes are tissue-specific or tissue-shared is lacking.

**Table 1:** Overview of studies of age-related DNA methylation changes in multiple tissues

Species	Tissues (*n*)	CpGs (Platform)	Comparison of overlap at each level			Ref
			CpG	Functional genomic region	Gene	
*Humans*						
Human	Buccal (96), liver (147), kidney (171) Th cells (214), brain (603), SC fat (648), monocytes (1202)	428,279 (Illumina 450 k)	+	+	+	Current study
Human	Cervix (3), bladder (5), intestine (5), kidney (6), head/neck (11), brain (12), pleura (18), placenta (19), lung (49), blood (85)	1413 (GoldenGate)	–	–	–	[21]
Human	Muscle (51), blood (71), brain (78), kidney (83)	26,486 (27 k array)	+	–	– (GO terms)	[22]
Human	Neuron (29), glia (29), MSCs (92), whole blood (656)	429,789 (Illumina 450 k)	+	–	–	[13]
*Rodents*						
Rat	Fat (3), liver (5–6)	40,000 (HELP assay)	–	–	–	[45]
Mouse	Liver (15), heart (15), lung (16), cortex (16)	1,230,000 (RRBS)	+	–	– (GO terms)	[46]

Here, we report on a systematic genome-wide analysis of age-related DNA methylation changes in a collection of 2858 methylomes from 7 tissues and show that the DNA methylation changes are highly tissue-specific and cannot be attributed to differences in statistical power. This tissue-specificity is not restricted to the individual CpG site but extends to the level of the functional region and the nearest gene to which a CpG maps. However, in every tissue, it is the same functional region (non-CGI, CGI, polycomb binding site, etc.) that accumulates age-related changes albeit at distinct locations in the genome.

## Results

**Fig. 1:**
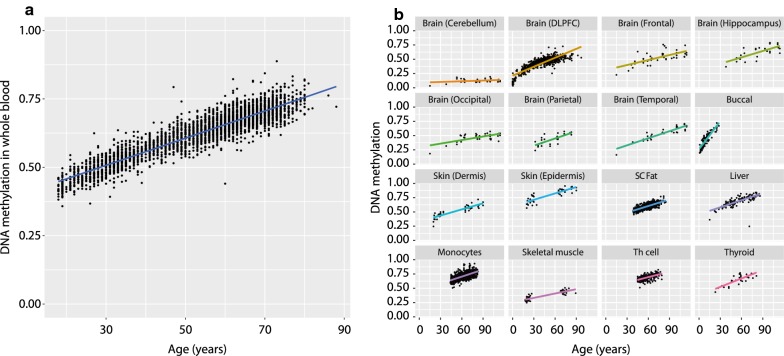
Age-related change in DNA methylation in *ELOVL2*. **a** DNA methylation (*y* axis) against age (*x* axis) in blood for the *ELOVL2* CpG (*cg16867657*). **b** DNA methylation (*y* axis) against age (*x* axis) in other tissues for the *ELOVL2* CpG (*cg16867657*)

To investigate age-related DNA methylation changes between tissues, Illumina 450k DNA methylation data were obtained from public repositories for 16 tissues encompassing in total 8092 individuals (Additional file 1: Table S1). First, we revisited the age-related differentially methylated position (aDMP) near *ELOVL2* (*cg16867657*), to test whether the tissue-independent character of this aDMP extended to multiple tissues. Gain of methylation was observed in blood (*N *= 3295, Fig. 1a) and extended to all tissues investigated except cerebellum (Fig. 1b) in line with previous reports [15].

**Fig. 2:**
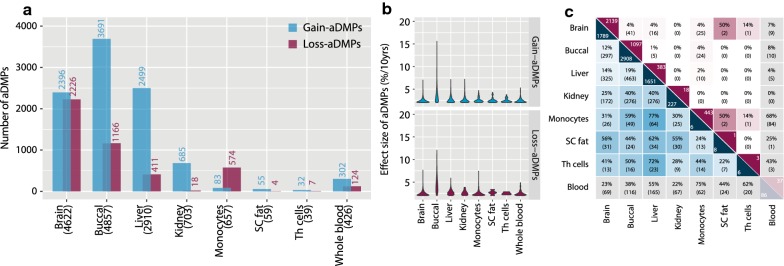
Identification of aDMPs. **a** Number of identified gain- and loss-aDMPs (*y* axis) in this study for each tissue (*x* axis). **b** Slopes of identified gain- and loss-aDMPs (*y* axis) for each tissue (*x* axis). **c** Overlap between tissues in identified gain- and loss-aDMPs. In the diagonal cells the number of aDMPs unique for that tissue, the upper number represents the percentage, the lower number the number of overlapping aDMPs. Blue—gain-aDMPs; Purple—loss-aDMPs

To systematically study the occurrence of tissue-specific and tissue-shared aDMPs, we identified aDMPs in tissues for which a moderate to large sample size was available (96 ≤ *N*≤1202; Additional file 1: Table S1), which included brain (*N* = 380), buccal (*N* = 96), liver (*N* = 147), kidney (*N* = 171), subcutaneous fat (SAT, *N* = 648), monocytes (*N* = 1202) and T-helper cells (Th cells, *N* = 214). As a comparison, whole-blood aDMPs were obtained from our previous work (*N* = 3295, [23]). We focused on a conservative set of aDMPs defined by genome-wide significance (*P*_bonf_ ≤ 0.05) and a robust age-related gain or loss that was larger than 2% per 10 years. Out of the 428,279 CpGs investigated, 7850 unique CpGs gained DNA methylation in one or more tissues (gain-aDMPs) and 4287 unique CpGs lost DNA methylation in one or more tissues (loss-aDMPs). The number of aDMPs identified in each tissue varied strongly, with the highest number in buccal (4857 aDMPs in *N* = 96; Fig. 2a, Additional File 2: Table S2) and the lowest number in Th cells (39 aDMPs in *N* = 214, Additional File 2: Table S2). As expected, whole-blood showed a substantial overlap with monocytes (62 gain-aDMPs, 84 loss-aDMPs, Additional File 2: Table S2) and Th cells (20 gain-aDMPs, 3 loss-aDMPs, Additional File 2: Table S2). Therefore, whole blood was not included in subsequent comparative analyses. The number of gain- versus loss-DMPs differed between tissues. For example, in liver, aDMPs mainly gained DNA methylation (gain 2499, loss 411; Fig. 2a, Additional File 2: Table S2), while in monocytes aDMPs mainly lost DNA methylation (gain 83, loss 574, Additional File 2: Table S2). Not only the number of DMPs but also the rates of change with age varied between tissues (Fig. 2b). The differences in number per tissue was not explained by either the known replication rate of the stem cells of the tissues analyzed (*r* = − 0.05, *P *= 0.90; Additional file 3: Fig. S1A) or the number of individuals used in each tissue (*r* = − 0.47, *P *= 0.24; Additional file 3: Fig. S1B).

### The majority of aDMPs are tissue-specific

**Fig. 3:**
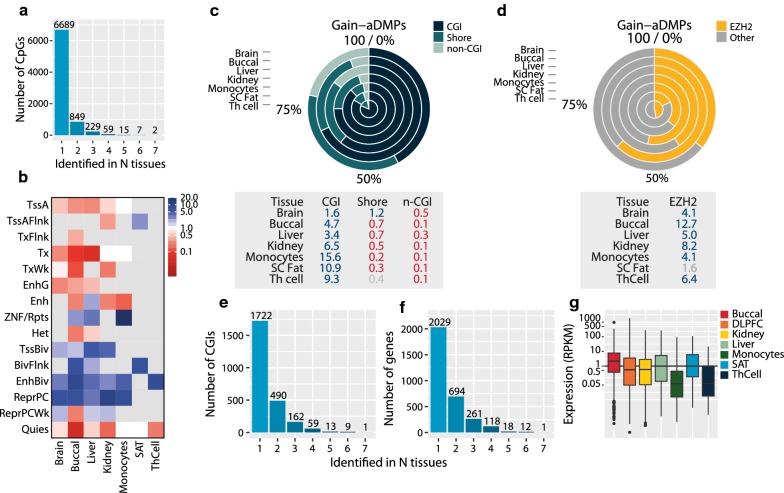
Characterization of gain-aDMPs. **a** Frequency of aDMPs (*y* axis) against the number of tissues the aDMPs was identified in (*x* axis). **b** Enrichment of gain-aDMPs in chromatin segmentations expressed as an odds ratio, grey non-significant. **c** Percentage (top) and odds ratios (bottom) of aDMPs in CGIs, shores and non-CGIs. Blue enriched, red depleted, grey non-significant. **d** Percentage (top) and odds ratios (bottom) of aDMPs in EZH2 binding sites (ChIP-seq, any cell type, ENCODE). Blue enriched, red depleted, grey non-significant. **e** Frequency of CpG islands (*y* axis) against the number of tissues a CpG island was identified in (*x* axis). **f** Frequency of genes (*y* axis) against the number of tissues a gene was identified in (*x* axis). **g** Expression (*y* axis, RPKM) of genes near gain-aDMPs per tissue (*x* axis). *Abbreviations*: *TssA* active TSS, *TssAFlnk* flanking active TSS, *TxFlnk* transcr. at gene 5′ and 3′, *Tx* strong transcription, *TxWk* weak transcription, *EnhG* genic enhancers, *Enh* enhancers, *ZNF/Rpts* ZNF genes + repeats, *Het* heterochromatin, *TssBiv* bivalent/poised TSS, *BivFlnk* flanking bivalent TSS/Enh, *EnhBiv* bivalent enhancer, *ReprPC* repressed polycomb, *ReprPCWk* weak repressed polycomb, *Quies* quiescent/low

The comparison of aDMPs between tissues showed that the large majority of aDMPs were tissue-specific (85.2% for gain-aDMPs and 97.4% for loss-aDMPs). Albeit low, the number of aDMPs shared between multiple tissues was higher for gain-aDMPs than for loss-aDMPs (Fig. 2c). Of the gain-aDMPs, 1161 (14.8%) were identified in ≥ 2 tissues (Fig. 3a). Only 2 gain-aDMPs were found in all 7 tissues studied and both mapped to the *ELOVL2* locus (Additional file 4: Table S3), thus underscoring that the strong tissue-shared association of these CpGs with age is exceptional. Loci consistently identified in blood [3] were also found in a subset of the tissues, that is 971 aDMPs (8.0%) overlapped with the 7477 CpGs previously identified in blood including *FHL2* (5 tissues) and *PENK* (4 tissues).

To exclude that the tissue-specificity was only due to the differences in size of the datasets, we performed three additional analyses. First, we identified aDMPs based on an effect size criterion only, thus eliminating the effect of statistical power. While CpGs in each of the tissues have an equal chance to become an aDMP, tissue-specificity was again observed in each of the tissues in the 37,136 aDMPs identified (brain 4786, buccal 27,884, liver 4225, kidney 3694, monocytes 769, SAT 222, Th cells 721; Additional file 5: Fig. S2A). Secondly, we identified aDMPs in equally sized datasets (*N* = 96, based on smallest tissue datasets) with both a *P* value (*P*_bonf_ ≤ 0.05) and effect size cut-off (> 2%/10 years) criterion. This approach resulted in 10,249 aDMPs across the 7 tissues (brain 4195, buccal 4857, liver 1636, kidney 499, monocytes 109, SAT 23), and these aDMPs were equally tissue-specific between tissues (Additional file 5: Fig. S2B). In these equally sized datasets, we observed that the aDMPs identified in one tissue were significant in other tissues at a less stringent *P* value cut-off (*P* < 0.001), suggesting that there may be a weak aDMP effect. However, this effect was much weaker as compared to the tissue the aDMP was identified in (Additional file 5: Fig. S2A–B, Additional file 6: Fig. S3).

Thirdly, the tissue-specificity of aDMPs was confirmed when we determined the age-related slope of the set of originally identified aDMPs in the all available 16 tissues (Additional file 6: Fig. S3). Together, these analyses reinforced the interpretation that aDMPs are truly tissue-specific and not due to differences in statistical power to detect aDMPs between tissues. Hence, age-related methylation changes occurring in one tissue are generally not indicative of age-related changes at the same CpGs in another tissue.

Identified aDMPs showed little overlap with the 353 CpGs from Horvath’s age predictor [24] (Additional file 7: Fig. S4). The maximum overlap with gain-aDMPs was found in brain (13 gain-aDMPs) and with loss-aDMPs in monocytes (6 loss-aDMPs). This is not unexpected, given that Horvath’s age predictor was trained using a penalized regression method aimed at identifying a sparse set of independent predictors and the fact that it was based on the Illumina 27k array (and hence did for example not include the *ELOVL2* CpGs). When Horvath’s tissue-independent age predictor was applied to the compendium of 16 tissues, the correlation between chronological age and predicted age was high although the precision of the prediction for individual samples was often limited (Additional file 8: Fig. S5). Only a minority of the CpGs that were included in Horvath’s age predictor show a strong association with age and the strongest tissue-specific aDMPs are missing (Additional file 9: Fig. S6). This illustrates the potential value of tissue-specific analyses to gain insight into the mechanisms linking age-related DNA methylation changes with tissue-specific ageing.

### Gain-aDMPs are tissue-specific but share their functional annotation

Genomic annotation showed that gain-aDMPs were highly enriched at CpG islands (CGIs) and their shores as compared with non-CGI sequences in each of the seven tissues (OR 1.6–15.6, *P *< 0.0001, Fig. 3c) and also in whole blood (OR 17.5, *P *< 0.0001, Additional file 10: Fig. S8A). These findings are in line with previous findings [1, 16, 25]. Utilizing reference chromatin segmentation data (marking the biological function of genomic regions) of primary tissues matching the tissues studied here [26], we found that gain-aDMPs preferentially occur at *Bivalent Enhancers* (OR 2.8–8.0, *P *< 0.0001, Fig. 3b) and *Repressed Polycomb* (3.4–9.8, *P *< 0.0001), both characterized by the polycomb repression mark H3K27me3. The latter observation was confirmed by the frequent co-occurrence of gain-aDMPs with binding sites of polycomb (PcG) repressive complex 2 (PRC2) protein EZH2. At least one-third of the identified gain-aDMPs overlapped with an EZH2 binding site increasing to almost two-thirds for buccal cells (OR 12.7, *P *< 0.0001, Fig. 3d). To address whether the enrichments for CGIs and EZH2 binding are independent or reflect the same underlying biology, we analysed both annotations together. Gain-aDMPs were twofold enriched (1.9–2.8, *P *< 0.0001) at genomic regions that were both CGIs and binding EZH2 as compared with regions that featured only one of the annotations (Additional file 11: Fig. S7A). This suggests that gain-aDMPs primarily occur at regions are both polycomb-repressed and CGIs. Finally, when the same enrichment analyses were performed on the whole-blood aDMPs, similar results were observed as compared to the other seven tissues (Additional file 10: Fig. S8A–C).

### CGIs and genes that gain methylation are also tissue-specific

Our analysis showed that, although individual gain-aDMPs are tissue-specific, their genomic annotation is shared. This was not due to different CpGs in the same CGI being identified as aDMPs across different tissues. This would go against the interpretation that gain-aDMPs are mainly tissue-specific. However, this was not the case. Of the 1,722 CGIs harbouring at least one gain-aDMP in at least one tissue, 70.1% were unique (Fig. 3e). The tissue-specificity further extended towards genes: mapping gain-aDMPs to their nearest gene, resulting in 2029 genes that were unique for a tissue (64.8%). Only one gene was found in all 7 tissues, namely (as expected) the *ELOVL2* gene (Fig. 3f and Additional file 4: Table S3). The 12 genes that were identified in 6 out of 7 tissues included *BMI1* (involved in the DNA damage response) and *LIN28B* (a microRNA that enhances IGF-2 translation). Counting the number of gain-aDMPs near a gene per tissue corroborated the tissue-specificity of genes. Genes with > 5 gain-aDMPs in one tissue had few in other tissues (Additional file 12: Table S4). Examples were *PRRT1* in the brain (brain 24, buccal 5, liver 7, kidney 1, monocytes 0, SAT 1, Th cell 2) and *HOXD* in buccal cells (buccal 26, other tissues 0).

Next, we investigated the function of genes near gain-aDMPs. In brain (84 processes), buccal (151 processes), liver (64 processes) and kidney (59 processes), multiple biological processes were enriched among nearest genes (*P*_bonf_ ≤ 0.05). Commonly and strongly enriched processes included *embryonic morphogenesis* (number of genes in brain 82, buccal 98, liver 69, kidney 37; *P*_bonf_ < 0.0001, Additional file 13: Table S5) and *regulation of transcription* (number of genes in brain 231, buccal 318, liver 209, kidney 134; *P*_bonf_ < 0.0001, Additional file 13: Table S5).

Finally, we investigated the expression of genes near gain-aDMPs using public gene expression data on tissues matching those studied here (GTEX data, frontal cortex, *N* = 108; oesophagus–mucosa, *N* = 286; liver, *N* = 119; kidney cortex, *N* = 32; whole blood, *N* = 393; age range 20–79 years). The baseline expression of genes was low (in line with their repressed state and developmental function), and we did not observe evidence for changes in gene expression (Fig. 3g and Additional file 11: Fig. S7B). Furthermore, there was little overlap between these genes and those previously reported to have a changed expression with age in whole blood (brain 91, buccal 104, liver 88, kidney 28, monocyte 1, SAT 3, Th cell 0) [27].

### Loss-aDMPs are enriched for active regions including tissue-specific enhancers

**Fig. 4:**
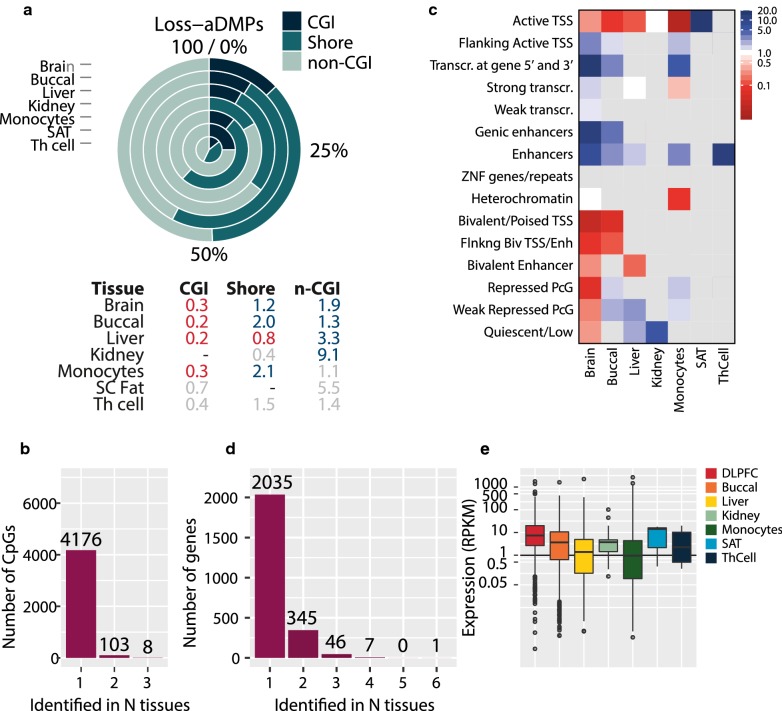
Characterization of loss-aDMPs. **a** Percentage and odds ratios of aDMPs in CGIs, shores and non-CGIs. Blue enriched, red depleted, grey non-significant. **b** Number of tissues an aDMPs was identified in. **c** Enrichment of gain-aDMPs in chromatin segmentations expressed as an odds ratio, grey non-significant enrichment. **d** Frequency of genes (*y* axis) against the number of tissues genes were identified in (*x* axis). **e** Expression of genes (*y* axis, RPKM) near loss-aDMPs per tissue (*x* axis). *Abbreviations*: *TssA* active TSS, *TssAFlnk* flanking active TSS, *TxFlnk* transcr. at gene 5′ and 3′, *Tx* strong transcription, *TxWk* weak transcription, *EnhG* genic enhancers, *Enh* enhancers, *ZNF/Rpts* ZNF genes + repeats, *Het* heterochromatin, *TssBiv* bivalent/poised TSS, *BivFlnk* flanking bivalent TSS/Enh, *EnhBiv* bivalent enhancer, *ReprPC* repressed polycomb, *ReprPCWk* weak repressed polycomb, *Quies* quiescent/low

In contrast to gain-aDMPs, loss-aDMPs preferentially occurred in non-CGI regions (OR 1.3–9.1, *P *< 0.0001, Fig. 4a) in the seven tissues and in whole blood (Additional file 10: Fig. S8D), in line with previous reports [1, 16, 25]. Loss-aDMPs were even more tissue-specific than gain-aDMPs: 4,176 loss-aDMPs (97.4%) were unique for one tissue and only 111 loss-aDMPs (2.6%) were found in ≥ 2 tissues (Fig. 4b). Again, contrasting with gain-aDMPs, loss-aDMPs were particularly overrepresented at chromatin states marking active genomic regions (Fig. 4c). In 5 of 7 tissues, an enrichment was found for *Enhancers* including in brain (OR 6.6, *P *< 0.0001, Fig. 4c), buccal cells (OR 2.7, *P *< 0.0001), liver (OR 1.6, *P *< 0.001), monocytes (OR 2.9, *P *< 0.0001) and Th cells (OR 11.2, *P *< 0.001). For 2 tissues, an enrichment for *Genic enhancers* was observed including brain (OR 11.9, *P *< 0.0001) and buccal (OR 3.6, *P *< 0.0001). Moreover, loss-aDMPs were overrepresented at actively transcribed regions, including *Transcribed at 3′and 5′* in brain (OR 14.5, *P *< 0.0001), buccal (OR 3.0, *P *< 0.0001) and monocytes (OR 4.6, *P *< 0.01). Again, these observations were comparable with enrichments for aDMPs in whole blood (Additional file 10: Fig. S8E).

Mapping loss-aDMPs to their nearest gene showed that the majority of genes uniquely occurred in a single tissue (2035 genes, 83.6%, Fig. 4d). The relatively few genes found in multiple tissues included *CD46* observed in 6 out of 7 tissues and *KCNQ1*, *FAM92B*, *PLEC*, *GSE1*, *BAIAP2*, *PRDM16* and *ACTG1* found in 4 tissues (Additional file 14: Table S6). Many of these genes have a ‘housekeeping’ function. For example, *PLEC, BAIAP2, ACTG1* play a role in the maintenance of the cytoskeleton. The tissue-specificity of loss-aDMP genes was corroborated when counting the number of loss-aDMPs per gene. For example, 24 loss-aDMPs were identified near *DIP2C* in the brain, against low numbers in other tissues (buccal 1, liver 3, kidney 0, monocytes 0, SAT 0, Th cell 0). In buccal, 18 CpGs lost DNA methylation near *SLC7A5*, while no loss-aDMPs were found near this gene in other tissues (Additional file 15: Table S7).

Only loss-aDMP genes in brain showed enrichment for specific biological processes, including regulation of *Small GTPase-mediated signal transduction* (43 genes, *P*_bonf_ < 0.0001) and *Regulation of cell motion* (33 genes, *P*_bonf_ < 0.05, Additional file 16: Table S8). For the other tissues, similar processes related to intracellular signalling and cell motility were overrepresented (*P *< 0.05).

Finally, we investigated the expression of genes near loss-aDMPs. Their expression levels were moderate (Fig. 4e), but no evidence was observed for age-related changes (Additional file 11: Fig. S7C). Also, only a limited overlap was found with previously identified age-related differentially expressed genes in whole blood (brain 114; buccal 77, liver 23, kidney 3, monocyte 36, SAT 0, Th cell 1) [27].

## Discussion

Using genome-wide DNA methylation data on a large number of individuals and 16 tissues, we report a catalogue of 7850 robust aDMPs, 92% of which had not been previously reported in studies of whole blood, and show that age-related changes in DNA methylation are highly tissue-specific. The exceptions to this are well-known CpGs in the *ELOVL2* promoter that display an exceptionally consistent increase in DNA methylation with age in all tissues studied here [15]. Age-related gain of DNA methylation (gain-aDMPs) accumulated at CpG islands and their flanking regions that were bound by the repressive PRC2 component EZH2. In contrast, loss-aDMPs were enriched for active regions, including enhancers. Underscoring the tissue-specificity of aDMPs, we observed that the large majority of both CGIs and genes with at least one aDMP were observed in a single tissue only.

Our results raise the question of what mechanism drives the age-related DNA methylation changes. Despite the tissue-specificity of individual aDMPs, in all tissues it was the same type of functional genomic region that accumulated aDMPs. We were able to exclude differences in the number of stem cell divisions between tissues as a potential explanation. In line with our findings for gain-aDMPs, age-related changes at regions marked by polycomb have been found in many studies investigating blood [7, 9, 10, 28] and have been found in other species [17, 29]. A previously proposed explanation for the gain of DNA methylation in CpG islands is by loss of binding—or erosion—of the polycomb repressive complex 2 protein from the DNA (PRC2) [30]. CGI promoters of developmental genes that are expressed at low levels are kept in a repressive state primarily by the repressive complex PRC2 of which EHZ2 is a key component. Age-related loss of repression would allow DNA methyltransferases (DNMTs) to de novo methylate CGIs [30]. This explanation, however, requires a region and tissue-specific mechanism that renders a subset of regions more susceptible to age-related erosion of PRC2, which is currently unknown. While the genomic annotations that show age-related gain across tissues were the same, the actual loci were tissue-specific, suggesting common underlying mechanisms that link to tissue-specific age-related changes.

In contrast to gain-aDMPs, loss-aDMPs overlapped with active genomic regions, such as enhancers, corroborating earlier studies in whole blood and mesenchymal stem cells [10, 13, 27]. Despite their tissue-specificity, genes near loss-aDMPs were not enriched for features conveying a role in tissue-specific processes, but instead with generic processes such as intracellular signalling cascade and cell motility pathways in line with findings in whole blood [3].

Remarkably, we did not find evidence for age-related changes in expression of genes near aDMPs. This confirms previous studies that aDMPs, including *ELOVL2,* have limited functional consequences [3, 9]. An explanation for the consistent increase in DMPs near *ELOVL2* and other age-related DNA methylation changes could be due to underlying mitotic changes, although this has not been observed in previous studies suggesting other mechanisms driving age-related DNA methylation changes [31, 32]. In contrast to aDMPs, CpGs accumulating variability in the population with age (aVMPs) are commonly associated with gene expression changes and may be more informative for biological age [23]. Nonetheless, age-related epigenetic changes show resemblance with the changes seen in cancer and cellular senescence [28]. For example, the number of passages in vitro can be tracked based on the changes that occur at the DNA methylation level [33]. Moreover, cellular senescence is associated with hypermethylation of CGIs and flanking regions, while hypomethylation occurs at non-CGI features [34, 35]. Cancer is also characterized by hypermethylation of CpG islands and global hypomethylation [36, 37]. Here, we observed a higher fraction of CpGs to gain DNA methylation with time as compared with loss. However, this is likely due to the bias of the 450 k array towards CpG-rich regions. After all, in a previous study comparing whole-genome bisulphite sequencing DNA methylation data of newborn versus a centenarian observed a much higher fraction of CpGs to be hypomethylated in the centenarian than hypermethylated [25].

A limitation of our study is that not all datasets were equally sized. Larger datasets will have higher statistical power to detect aDMPs with smaller effect sizes. However, we showed that there was no relationship between sample size and the tissue-specific character of aDMPs as this was preserved if aDMPs were identified only based on effect size, in equally sized datasets, or in 16 instead of 7 tissues. Also, one would expect at least to find an overlap between the strongest associated aDMPs, but this was not the case. Inspection of effect sizes showed that CpGs detected as aDMPs in one tissue commonly showed little or no evidence for an age-related change in DNA methylation in other tissues. However, larger studies are required to definitely exclude smaller effects in other tissues.

Another limitation of our study is that the age ranges across the different tissues were different, which will have influenced the number of aDMPs detected. Age-related DNA methylation changes are known to accumulate faster during adolescence than in adulthood [38, 39]. This may have contributed the identification of the largest number of aDMPs in the smallest dataset, namely buccal (*n* = 96; age range 1–28). Finally, our results may be influenced by measured and unmeasured confounding, such as smoking, BMI, ethnicity and shifts in cell heterogeneity [7]. Some of the changes identified here may also be the result of a shift in cellular composition of a tissue with age, although we adjusted for cellular heterogeneity in brain (neuronal and non-neuronal), monocytes, Th cells (residual impurities) and blood.

## Conclusion

Together, our results show that while the individual CpGs that exhibit age-related differential methylation are highly tissue-specific, the type of functional genomic elements involved are highly consistent across tissues. Gain of methylation occurs at CGIs repressed by PRC2, while loss of methylation accumulates at regions with active histone marks. Our findings indicate that the precision of age predictors based on DNA methylation will depend on whether the tissue of interest was among the tissues on which set the predictor was trained on. Our catalogue of tissue-specific aDMPs may guide the development of more precise predictors of chronological and perhaps eventually provide insight into the tissue-specific differences in the mechanisms underlying ageing.

## Methods

### Datasets

Datasets used in this study are summarized in Additional file 1: Table S1 and were obtained from the Gene Expression Omnibus or ArrayExpress. For each of the datasets, normalized data or raw IDAT files were obtained. IDAT files of DLPFC samples (age range 0–97 years) were downloaded (GEO accession number: GSE74193). Initial QC was performed using the R package *MethylAid* [40]. Raw data underwent quality control using a custom pipeline (for more details see https://git.lumc.nl/molepi/Leiden450K). Briefly, data were normalized using functional normalization (*minfi*), and probes were set to missing if ambiguously mapped, had a high detection *P* value (> 0.01), low bead count (< 3 beads) or low success rate (missing in > 95% of the samples). Normalized data of buccal (GEO accession number: GSE50759, normalization: SWAN method on M values) consisted of 1202 individuals with an age range of 1–28 years. Liver data consisted of 147 individuals with age range between 15 and 86, normalized data of 56 individuals (GEO accession number: GSE48325, normalization using control probes), normalized data of 32 individuals (GEO accession number: GSE61258, normalization using control probes), IDAT files (Level 1) of 30 samples from TCGA and IDAT files of 29 samples were kindly provided by the authors (GSE60753). Given that the liver dataset consisted of dataset from different origins, we carefully inspected for batch effects influencing the age-related changes. The first principal components associated with study, sex and age. To limit the effect of data origin, study ID was added to the model. IDAT files (Level 1) of kidney consisted of 171 individuals with an age range between 15 and 86 and were obtained from TCGA. Normalized data of monocytes (GEO accession number: GSE56046, normalization: quantile normalization per colour signal and probe type) consisted of 1,202 individuals with an age range of 44–83 years. Normalized data of Th cells (GEO accession number: GSE56047, normalization: quantile normalization) consisted of 214 individuals with an age range of 45–79 years). Normalized data of subcutaneous fat (Array Expression accession number: E-MTAB-1866, quantile normalized per probe type) consisted of 648 individuals with an age range of 39–85 years. Normalized data of multiple brain regions consisted between 25 and 41 individuals with an age range between 15 and 114 years (GEO accession number: GSE64509, normalization using control probes). Raw IDAT files of epidermis and dermis consisted 38 and 40 individuals between 20 and 90 years (GEO accession number: GSE52980). Normalized data of skeletal muscle consisted of 48 individuals with an age range between 18 and 89 years (GEO accession number: GSE50498, normalization: quantile normalization on M values). Raw IDAT files of thyroid were kindly provided by the authors and consisted of 28 individuals between 23 and 81 years (GEO accession number: GSE53051).

#### Blood dataset

DNA methylation blood data consisted of 3,295 from six Dutch biobanks, previously described [23]. Briefly, data were normalized using functional normalization (*R* package minfi) using five principal components [41], poorly performing and ambiguously mapped CpGs were removed as well as CpGs on the sex chromosomes. Combat was used to remove residual batch effects [42].

#### Gene expression

Gene counts were obtained from GTEX for frontal cortex (for brain-aDMPs measured in DLPFC), oesophagus mucosa (for buccal-aDMPs), liver, kidney cortex and whole blood (for Th cell-aDMPs and monocyte-aDMPs). The package *cqn* was used to normalize for GC content and gene length. Normalized data was used to calculate the average RPKM per tissue and gene.

### Statistical analysis

aDMPs were identified using linear regression between DNA methylation and age, with adjustment for covariates (sex, gender (all but SAT, females only), dataset (liver), tissue cell composition (DLPFC, monocytes, Th cells, whole blood). For monocytes and Th cells, residual cell impurities were included in the model. For whole blood, blood cell fractions were included as previously described [23]. Age was included in the model as a non-transformed numeric variable. aDMPs were used in subsequent analyses if the slope was higher than 2% gain or loss per 10 years and if the Bonferroni adjusted *P* value reached significance (*P*_bonf_ ≤ 0.05). To investigate the relation between power and the observed tissue-specific character of aDMPs, we identified aDMPs solely based on the effect size (age-related slope > 2%/10 year, no *P* value cut-off). Secondly, a random set of individuals was drawn from each of the tissue datasets with the size equal to the smallest dataset (*N* = 96). aDMPs were identified on these equally sized datasets with both an effect size (> 2%/10 years) and *P* value criterion (*P*_bonf_ ≤ 0.05).

#### Annotations

CpGs were mapped to CpG islands (UCSC), shores (2-kb regions flanking regions) and non-CGI described previously [43]. Chromatin state segmentations were obtained from the Epigenomics Roadmap. For each tissue studied, the same tissue or the closest analogue was used from the Roadmap data. For the DLPFC, E073/DLPFC was used; buccal, E058/keratinocyte foreskin; liver, E066/liver; kidney, E086/foetal kidney; SAT, E063/Adipose nuclei; monocytes, E029/Monocytes; Th cells, E043/Th cells. For blood, functionality of a certain regions was based on the most frequent occurring feature in primary blood cell subtypes. ChIP-seq data of EZH2 was obtained for all cell types from the ENCODE project. Enrichments were expressed as odds ratio, and *P* value were calculated using a Chi-squared test. GO enrichment was performed using the default settings of DAVID using nearest genes (UCSC, 3′ or 5′ end of genes closest to the CpG) of aDMPs [44].

## Additional files

**Additional file 1: Table S1.** Number of individuals used per tissue in this study.

**Additional file 2: Table S2.** Identified aDMPs per tissue.

**Additional file 3: Figure S1.** **A** Number of aDMPs (*y* axis) in our study against the previously reported number of stem cell divisions per year (*x* axis) [47]. **B** Number of aDMPs (*y* axis) against the sample size (*x* axis).

**Additional file 4: Table S3.** Number of tissues a gene near gain-aDMPs was found.

**Additional file 5: Figure S2.** **A** Heatmap of slopes of aDMPs identified with only an effect size criterion. **B** Heatmap of slopes of aDMPs identified in equally sized datasets comprising randomly selected 96 individuals. Scale represents the change in DNA methylation in %/10 years. **C** Number of significant (*P* < 0.001) aDMPs in the other tissues in the equally-sized datasets.

**Additional file 6: Figure S3.** Heatmap of slopes of age-related DNA methylation in 16 tissues. Scale represents the change in DNA methylation in %/10 years.

**Additional file 7: Figure S4.** Overlap between gain- and loss-aDMPs and the CpGs in Horvath’s clock.

**Additional file 8: Figure S5.** Chronological age against the Horvath’s predicted age for each of the 16 tissues.

**Additional file 9: Figure S6.** Volcano plots of all CpGs per tissue, age-related change (*x* axis) versus *P* value (*y* axis). CpGs from Horvath’s age predictor are marked in blue.

**Additional file 10: Figure S8.** **A** Percentage (top) and odds ratios (bottom) of gain-aDMPs in CGIs, shores and non-CGIs. Blue enriched, red depleted, grey non-significant. **B** Percentage (top) and odds ratios (bottom) of aDMPs in EZH2 binding sites in the seven tissues plus whole blood (ChIP-seq, any cell type, ENCODE). Blue enriched, red depleted, grey non-significant. **C** Enrichment of gain-aDMPs in chromatin segmentations expressed in the seven tissues plus whole blood as an odds ratio, grey non-significant. **D** Percentage (top) and odds ratios (bottom) of loss-aDMPs in CGIs, shores and non-CGIs. Blue enriched, red depleted, grey non-significant. **E** Enrichment of loss-aDMPs in chromatin segmentations expressed in the seven tissues plus whole blood as an odds ratio, grey non-significant. Abbreviations: TssA, Active TSS; TssAFlnk, Flanking active TSS; TxFlnk, Transcr. at gene 5′ and 3′; Tx, Strong transcription; TxWk, Weak transcription; EnhG, Genic enhancers; Enh, Enhancers; ZNF/Rpts, ZNF genes + repeats; Het, Heterochromatin; TssBiv, Bivalent/Poised TSS; BivFlnk, Flanking bivalent TSS/Enh; EnhBiv, Bivalent enhancer; ReprPC, Repressed Polycomb; ReprPCWk, Weak repressed Polycomb, Quies, Quiescent/low.

**Additional file 11: Figure S7.** **A** Percentage (top) and enrichment (odds ratio, bottom) for CGI and the polycomb protein EZH2 and the combination. **B** Expression (*y* axis, RPKM) of genes near gain- and loss-aDMPs for each tissue for each age category (*x* axis).

**Additional file 12: Table S4.** Frequency of gain-aDMPs near genes.

**Additional file 13: Table S5.** Enriched GO terms for gain-aDMPs per tissue.

**Additional file 14: Table S6.** Number of tissues a gene near loss-aDMPs was found.

**Additional file 15: Table S7.** Frequency of loss-aDMPs near genes.

**Additional file 16: Table S8.** Enriched GO terms for loss-aDMPs per tissue.

## Abbreviations

aDMPs: age-related differentially methylated positions
BivFlnk: flanking bivalent transcription start site or enhancer
DLPFC: dorsolateral prefrontal cortex
Enh: enhancers
EnhBiv: bivalent enhancer
EnhG: genic enhancers
Het: heterochromatin
SAT: subcutaneous fat
Th cells: T helper cells
PcG: polycomb group
PRC2: polycomb repressive complex 2
Quies: quiescent/low
ReprPC: repressed polycomb
ReprPCWk: weak repressed polycomb
TssA: active TSS
TssAFlnk: flanking active TSS
TssBiv: bivalent/poised transcription start site
Tx: strong transcription
TxFlnk: transcribed at gene 5′ and 3′
TxWk: weak transcription
ZNF/Rpts: zinc fingers genes and repeats
