# Supplementary material for: Age-related DNA methylation changes are tissue-specific with ELOVL2 promoter methylation as exception
Source: Epigenetics Chromatin. 2018 May 30;11:25. doi: 10.1186/s13072-018-0191-3 (PMC5975493; doi:10.1186/s13072-018-0191-3)
Supplement: Supplementary file 1 — Additional file 1: Table S1. Number of individuals used per tissue in this study. [file 13072_2018_191_MOESM1_ESM.docx]

|  |  | **Age** |  |  | **Gender** | |  |  |
| --- | --- | --- | --- | --- | --- | --- | --- | --- |
| **Tissue** | **N** | **Median** | **Min** | **Max** | **M** | **F** | **GEO** | **Reference** |
| **Discovery data** |  |  |  |  |  |  |  |  |
| Monocytes | 1202 | 60 | 44 | 83 | 580 | 622 | GSE56047 | (Reynolds et al., 2014) |
| SC Fat | 648 | 60 | 39 | 85 | 0 | 648 | E-MTAB-1866 | (Grundberg et al., 2012) |
| Brain DLPFC | 380 | 35 | 0 | 85 | 123 | 257 | GSE74193 | (Jaffe et al., 2015) |
| Th cells | 214 | 58 | 45 | 79 | 106 | 108 | GSE56047 | (Reynolds et al., 2014) |
| Kidney | 171 | 63 | 39 | 90 | 113 | 58 | TCGA | (TCGA Research Network) |
| Liver | 147 | 54 | 15 | 86 | 58 | 89 | TCGA, GSE60753,  GSE48325, GSE61258 | (Ahrens et al., 2013; Hlady et al., 2014; Horvath et al., 2014; TCGA Research Network) |
| Buccal | 96 | 7 | 1 | 28 | 58 | 38 | GSE50759 | (Berko et al., 2014) |
|  |  |  |  |  |  |  |  |  |
| Brain CRBM | 32 | 85 | 15 | 114 | 11 | 21 | GSE64509 | (Horvath et al., 2015) |
| Brain Frontal | 41 | 80 | 15 | 114 | 19 | 22 | GSE64509 | (Horvath et al., 2015) |
| Brain Hippocampus | 25 | 85 | 38 | 114 | 8 | 17 | GSE64509 | (Horvath et al., 2015) |
| Brain Occipital | 33 | 85 | 15 | 114 | 11 | 22 | GSE64509 | (Horvath et al., 2015) |
| Brain Parietal | 23 | 53 | 32 | 83 | 19 | 4 | GSE64509 | (Horvath et al., 2015) |
| Brain Temporal | 29 | 82 | 15 | 114 | 8 | 21 | GSE64509 | (Horvath et al., 2015) |
| Dermis | 40 | 49.5 | 20 | 90 | 18 | 22 | GSE52980 | (Vandiver et al., 2015) |
| Epidermis | 38 | 65 | 20 | 90 | 17 | 21 | GSE52980 | (Vandiver et al., 2015) |
| Muscle Skel | 48 | 47.5 | 18 | 89.1 | 48 | 0 | GSE50498 | (Zykovich et al., 2014) |
| Thyroid | 28 | 57.5 | 23 | 81 | 13 | 15 | GSE53051 | (Timp et al., 2014) |
|  |  |  |  |  |  |  |  |  |
| **Whole blood datasets** |  |  |  |  |  |  |  |  |
| Blood | 3295 | 57 | 18 | 88 | 1413 | 1882 | GSE40279 | (Slieker et al., 2016) |
|  |  |  |  |  |  |  |  |  |
| **Expression data** |  |  |  |  |  |  |  |  |
| Frontal Cortex (BA9) | 108 | - | 20 | 79 | 77 | 31 | GTEX | (Lonsdale et al., 2013) |
| Esophagus - Mucosa | 286 | - | 20 | 79 | 179 | 107 | GTEX | (Lonsdale et al., 2013) |
| Liver | 119 | - | 20 | 79 | 78 | 41 | GTEX | (Lonsdale et al., 2013) |
| Kidney - Cortex | 32 | - | 20 | 79 | 25 | 7 | GTEX | (Lonsdale et al., 2013) |
| Whole blood | 393 | - | 20 | 79 | 249 | 144 | GTEX | (Lonsdale et al., 2013) |

Ahrens, M., Ammerpohl, O., von Schönfels, W., Kolarova, J., Bens, S., Itzel, T., Teufel, A., Herrmann, A., Brosch, M., and Hinrichsen, H. (2013). DNA methylation analysis in nonalcoholic fatty liver disease suggests distinct disease-specific and remodeling signatures after bariatric surgery. Cell metabolism *18*, 296-302.

Berko, E.R., Suzuki, M., Beren, F., Lemetre, C., Alaimo, C.M., Calder, R.B., Ballaban-Gil, K., Gounder, B., Kampf, K., and Kirschen, J. (2014). Mosaic epigenetic dysregulation of ectodermal cells in autism spectrum disorder. PLoS genetics *10*.

Grundberg, E., Small, K.S., Hedman, Å.K., Nica, A.C., Buil, A., Keildson, S., Bell, J.T., Yang, T.-P., Meduri, E., and Barrett, A. (2012). Mapping cis-and trans-regulatory effects across multiple tissues in twins. Nature genetics *44*, 1084-1089.

Hlady, R.A., Tiedemann, R.L., Puszyk, W., Zendejas, I., Roberts, L.R., Choi, J.-H., Liu, C., and Robertson, K.D. (2014). Epigenetic signatures of alcohol abuse and hepatitis infection during human hepatocarcinogenesis. Oncotarget *5*, 9425.

Horvath, S., Erhart, W., Brosch, M., Ammerpohl, O., von Schönfels, W., Ahrens, M., Heits, N., Bell, J.T., Tsai, P.-C., and Spector, T.D. (2014). Obesity accelerates epigenetic aging of human liver. Proceedings of the National Academy of Sciences *111*, 15538-15543.

Horvath, S., Mah, V., Lu, A.T., Woo, J.S., Choi, O.-W., Jasinska, A.J., Riancho, J.A., Tung, S., Coles, N.S., and Braun, J. (2015). The cerebellum ages slowly according to the epigenetic clock. Aging *7*.

Jaffe, A.E., Gao, Y., Deep-Soboslay, A., Tao, R., Hyde, T.M., Weinberger, D.R., and Kleinman, J.E. (2015). Mapping DNA methylation across development, genotype and schizophrenia in the human frontal cortex. Nat Neurosci *advance online publication*.

Lonsdale, J., Thomas, J., Salvatore, M., Phillips, R., Lo, E., Shad, S., Hasz, R., Walters, G., Garcia, F., and Young, N. (2013). The genotype-tissue expression (GTEx) project. Nature genetics *45*, 580-585.

Reynolds, L.M., Taylor, J.R., Ding, J., Lohman, K., Johnson, C., Siscovick, D., Burke, G., Post, W., Shea, S., Jacobs Jr, D.R.*, et al.* (2014). Age-related variations in the methylome associated with gene expression in human monocytes and T cells. Nat Commun *5*.

Slieker, R.C., van Iterson, M., Luijk, R., Beekman, M., Zhernakova, D.V., Moed, M.H., Mei, H., Van Galen, M., Deelen, P., and Bonder, M.J. (2016). Age-related accrual of methylomic variability is linked to fundamental ageing mechanisms. Genome biology *17*, 191.

TCGA Research Network. <http://cancergenome.nih.gov/>.

Timp, W., Bravo, H.C., McDonald, O.G., Goggins, M., Umbricht, C., Zeiger, M., Feinberg, A.P., and Irizarry, R.A. (2014). Large hypomethylated blocks as a universal defining epigenetic alteration in human solid tumors. Genome Med *6*, 61.

Vandiver, A.R., Irizarry, R.A., Hansen, K.D., Garza, L.A., Runarsson, A., Li, X., Chien, A.L., Wang, T.S., Leung, S.G., and Kang, S. (2015). Age and sun exposure-related widespread genomic blocks of hypomethylation in nonmalignant skin. Genome biology *16*, 80.

Zykovich, A., Hubbard, A., Flynn, J.M., Tarnopolsky, M., Fraga, M.F., Kerksick, C., Ogborn, D., MacNeil, L., Mooney, S.D., and Melov, S. (2014). Genome‐wide DNA methylation changes with age in disease‐free human skeletal muscle. Aging Cell *13*, 360-366.
