# Supplementary figures and images for: Age-related DNA methylation changes are tissue-specific with ELOVL2 promoter methylation as exception
Source: Epigenetics Chromatin. 2018 May 30;11:25. doi: 10.1186/s13072-018-0191-3 (PMC5975493; doi:10.1186/s13072-018-0191-3)

**A**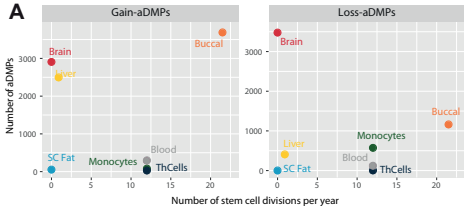**B**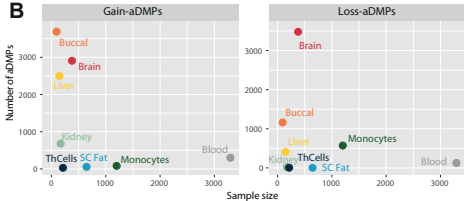

Supplement: Supplementary file 3 — Additional file 3: Figure S1. A Number of aDMPs (y axis) in our study against the previously reported number of stem cell divisions per year (x axis) [47]. B Number of aDMPs (y axis) against the sample size (x axis). [file 13072_2018_191_MOESM3_ESM.pdf]

# Slope with age (%/10yrs)

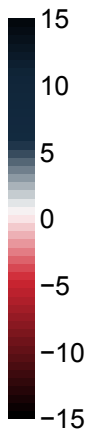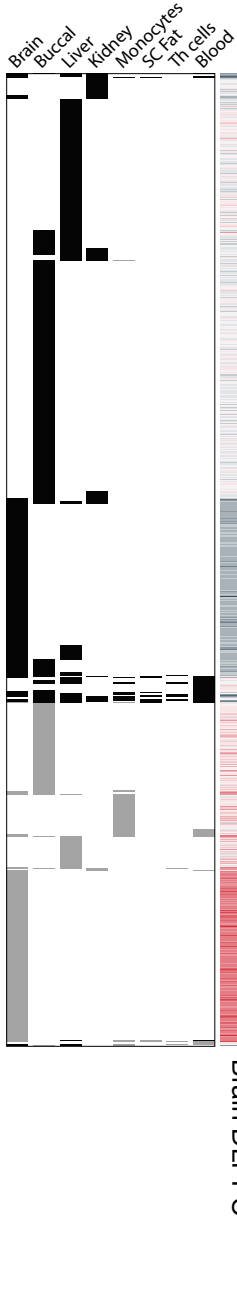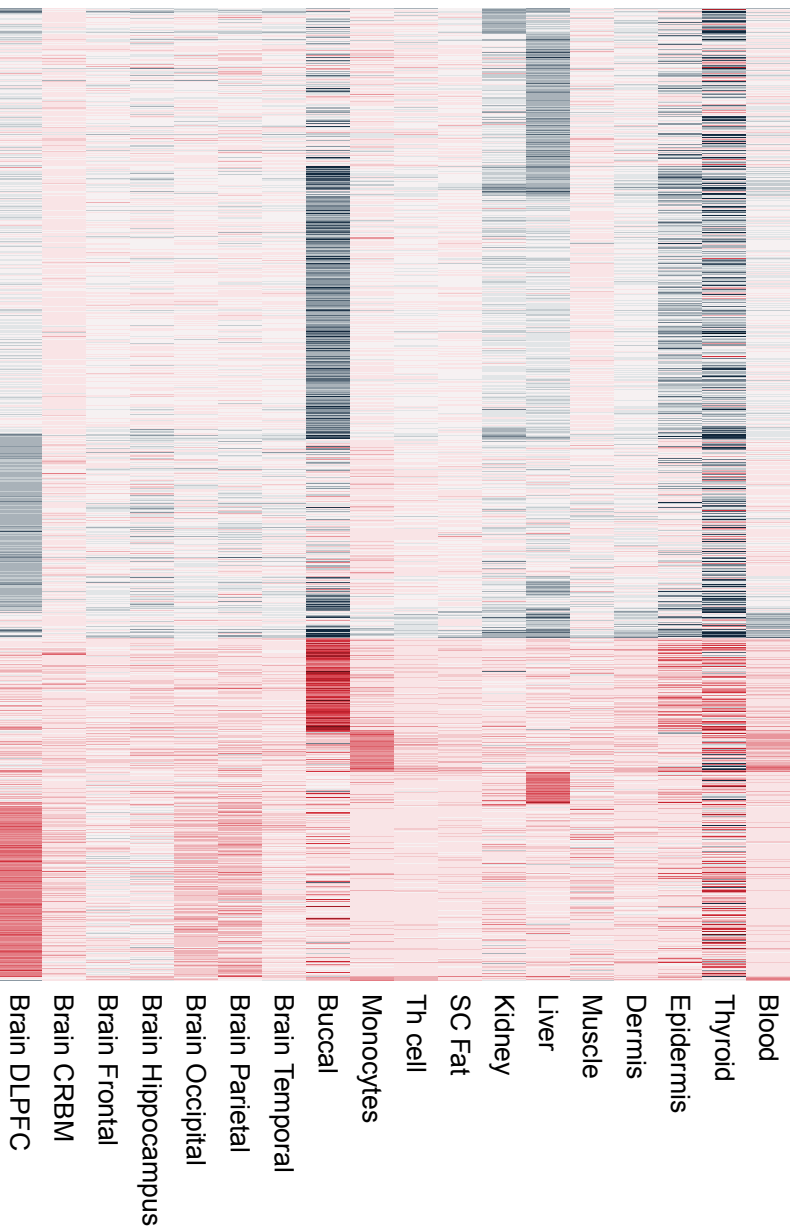

Supplement: Supplementary file 6 — Additional file 6: Figure S3. Heatmap of slopes of age-related DNA methylation in 16 tissues. Scale represents the change in DNA methylation in %/10 years. [file 13072_2018_191_MOESM6_ESM.pdf]

## Gain-aDMPs

## Loss-aDMPs

Brain

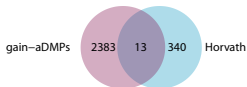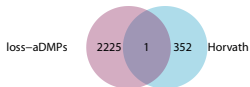

Buccal

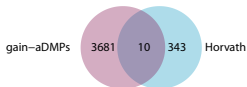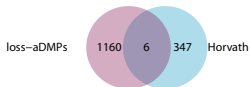

Liver

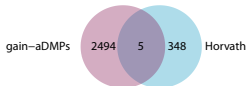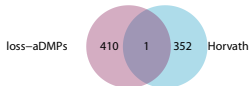

Kidney

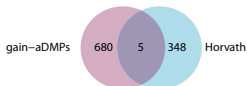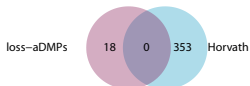

Monocytes

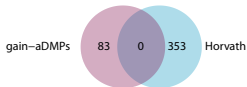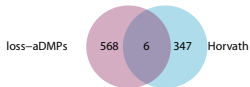

SAT

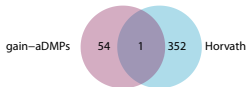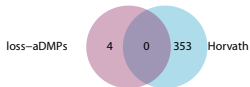

ThCell

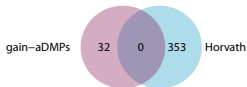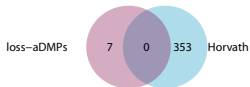

Blood

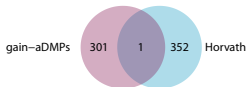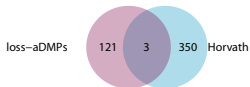

Supplement: Supplementary file 7 — Additional file 7: Figure S4. Overlap between gain- and loss-aDMPs and the CpGs in Horvath’s clock. [file 13072_2018_191_MOESM7_ESM.pdf]

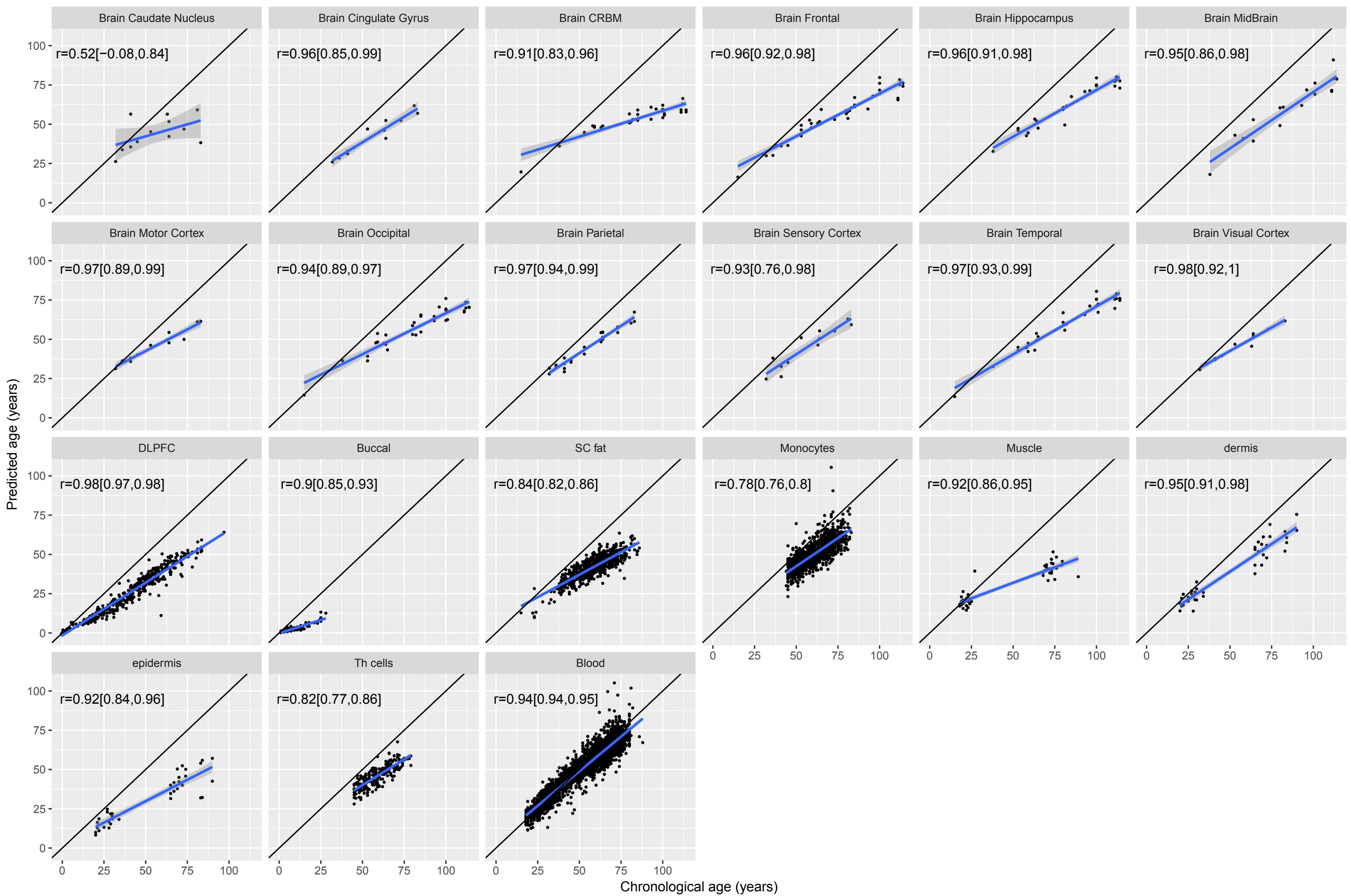

Supplement: Supplementary file 8 — Additional file 8: Figure S5. Chronological age against the Horvath’s predicted age for each of the 16 tissues. [file 13072_2018_191_MOESM8_ESM.pdf]

DLPFC

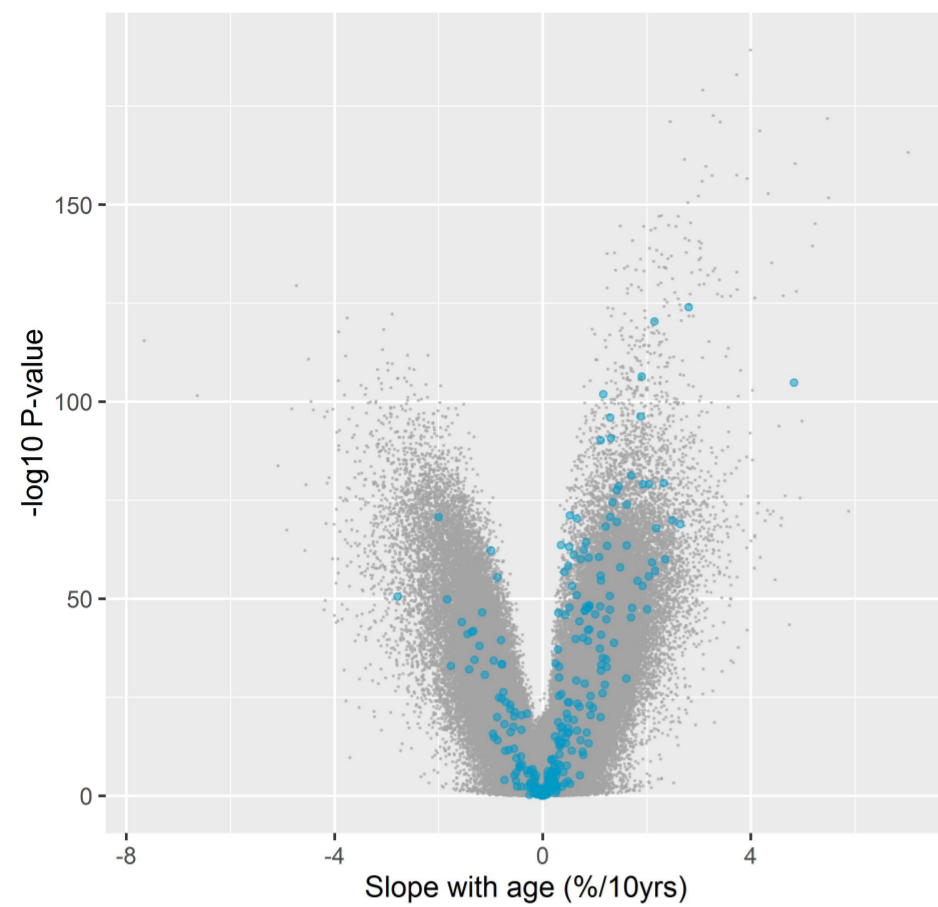

Buccal

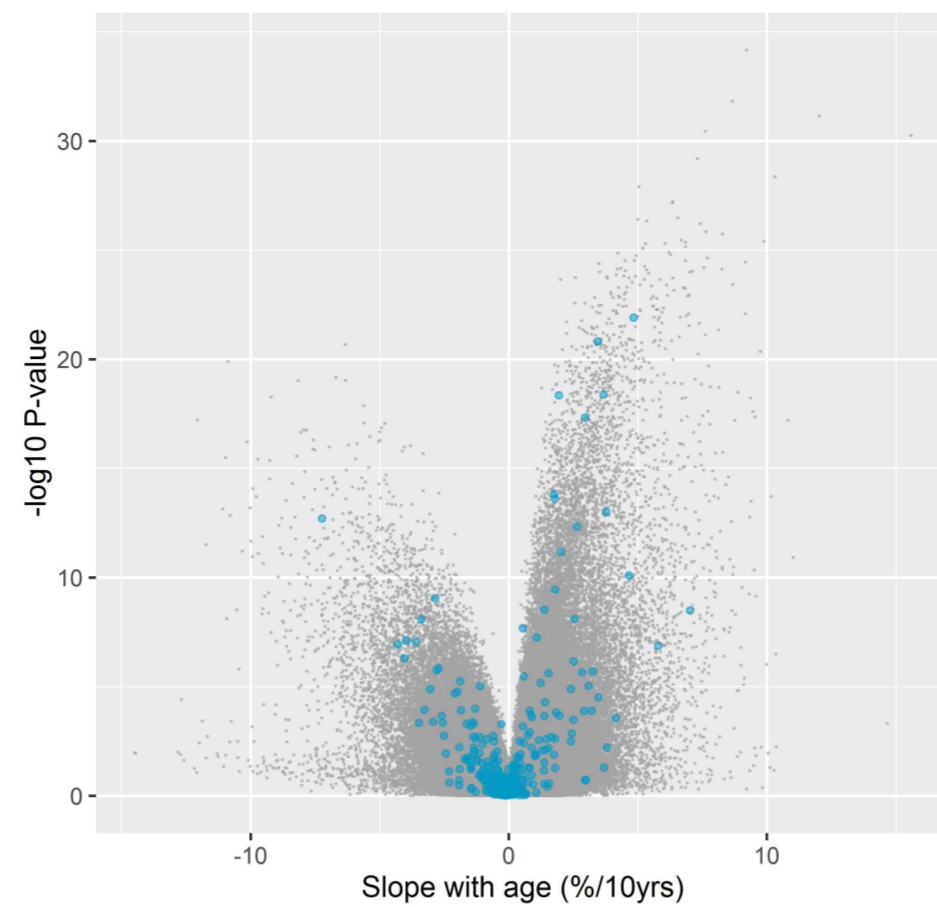

Liver

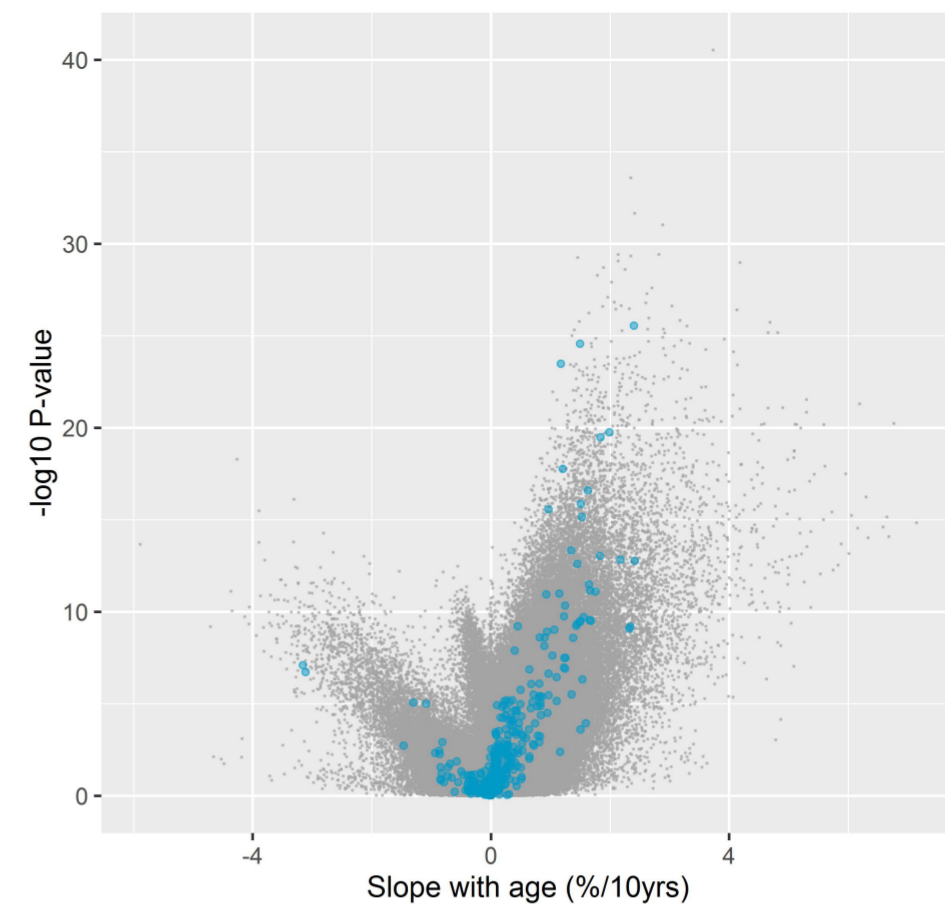

Kidney

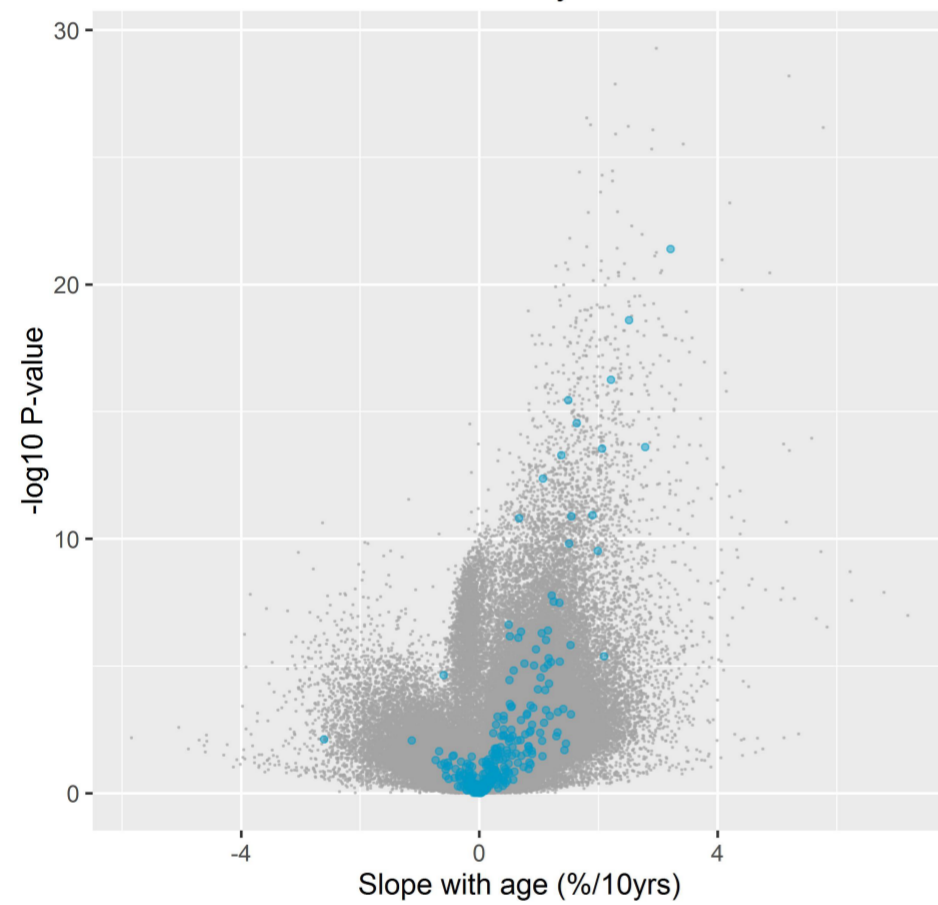

Monocytes

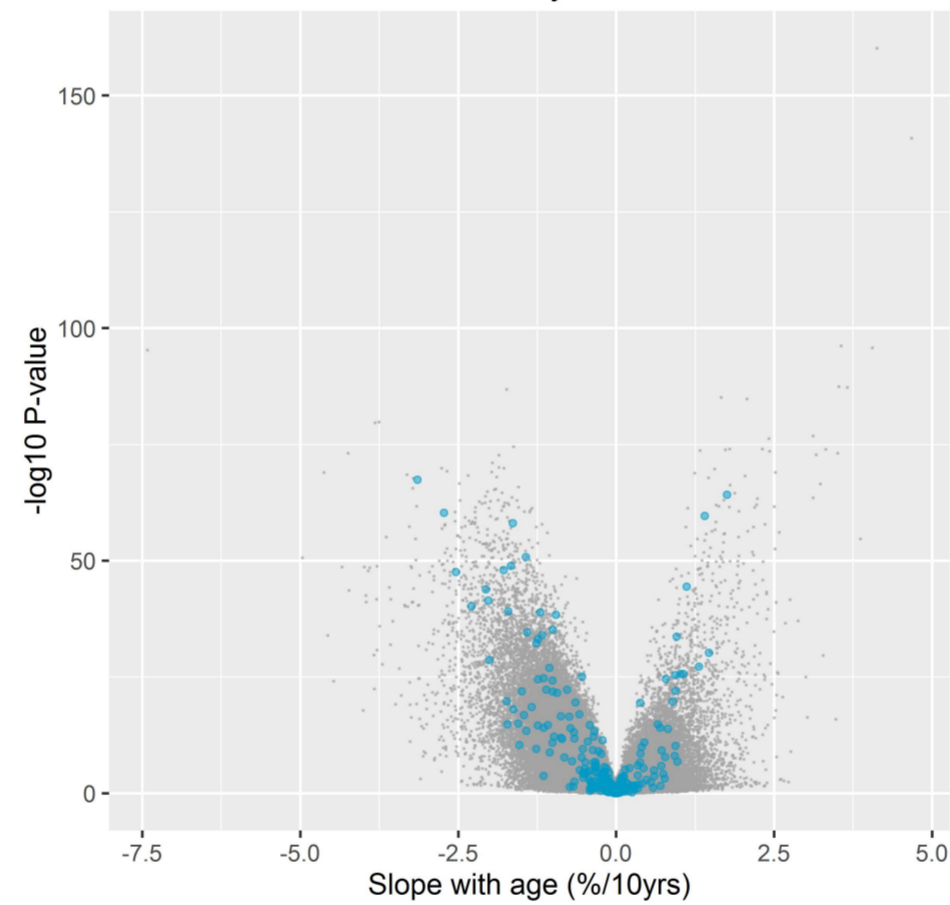

SCfat

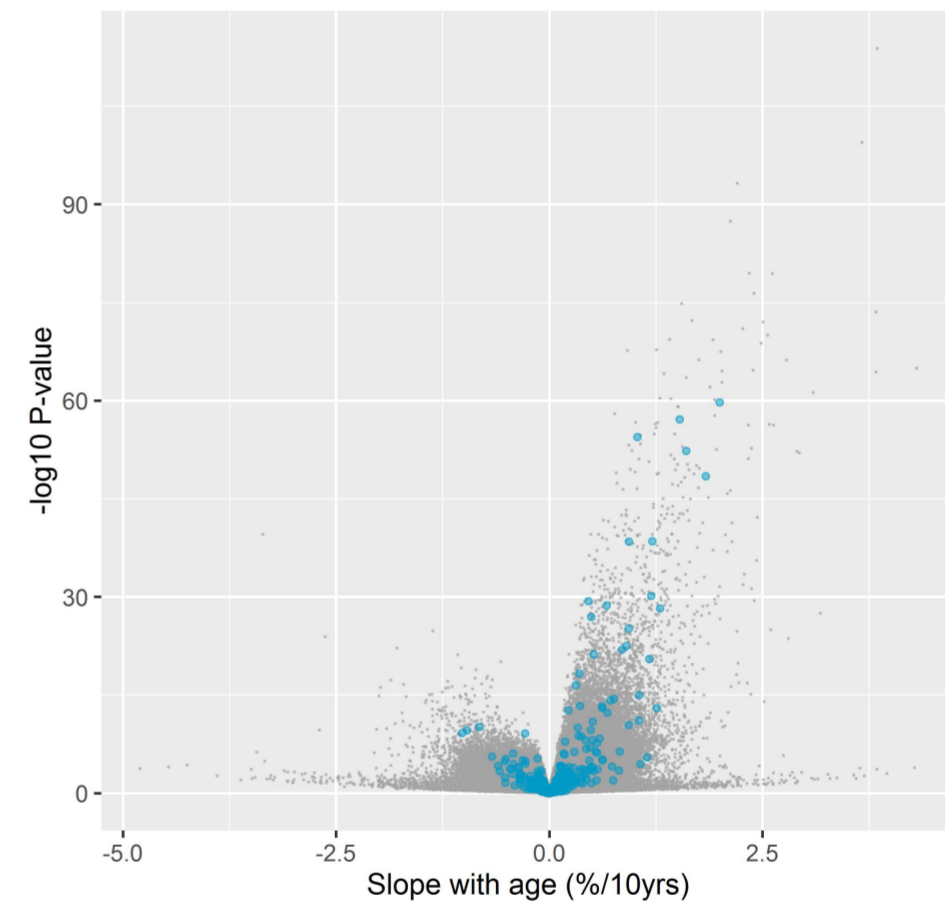

ThCell

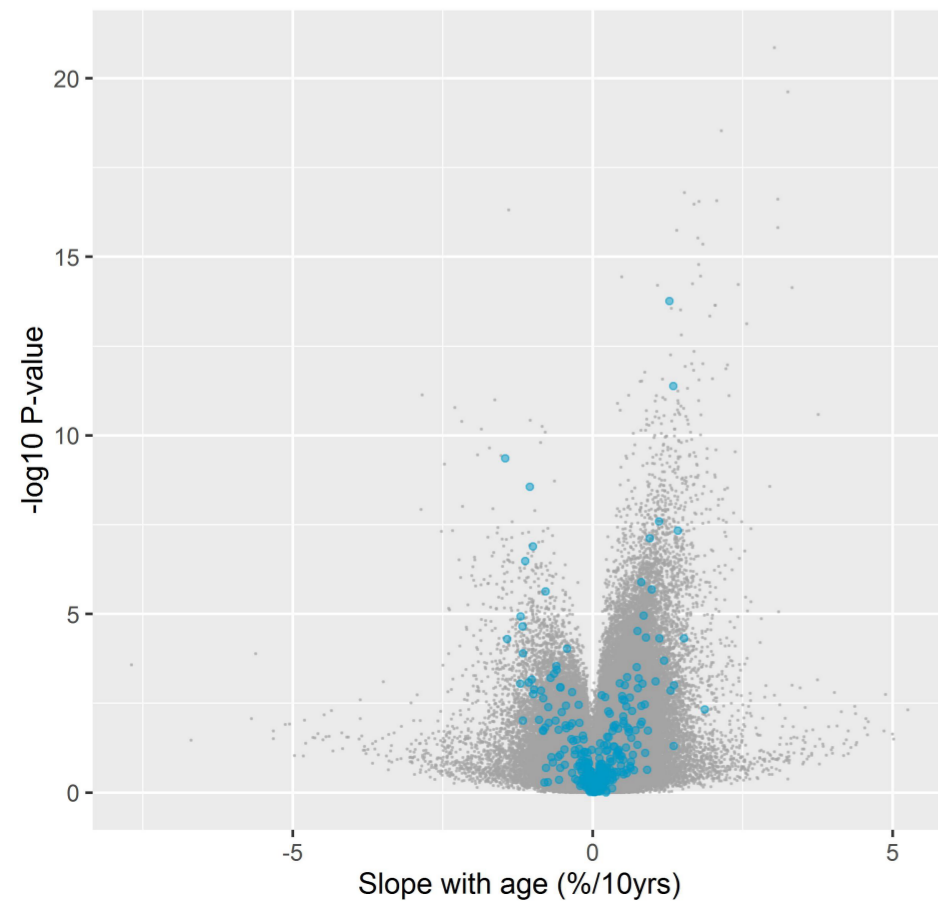

Blood

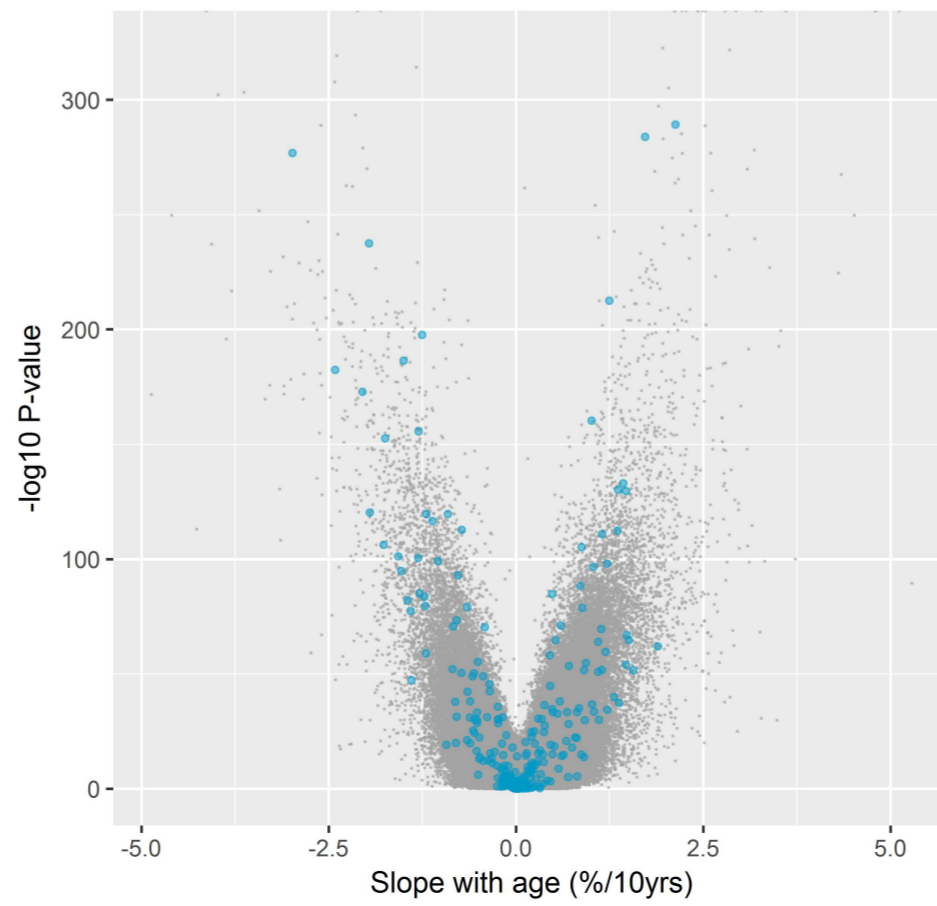

Supplement: Supplementary file 9 — Additional file 9: Figure S6. Volcano plots of all CpGs per tissue, age-related change (x axis) versus P value (y axis). CpGs from Horvath’s age predictor are marked in blue. [file 13072_2018_191_MOESM9_ESM.pdf]

**A**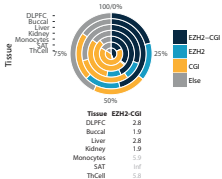**B**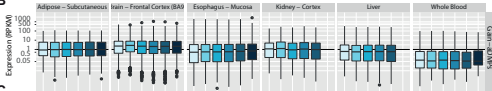**C**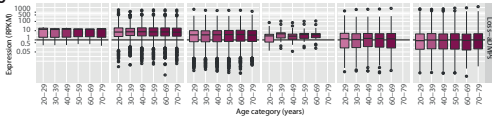

Supplement: Supplementary file 11 — Additional file 11: Figure S7. A Percentage (top) and enrichment (odds ratio, bottom) for CGI and the polycomb protein EZH2 and the combination. B Expression (y axis, RPKM) of genes near gain- and loss-aDMPs for each tissue for each age category (x axis). [file 13072_2018_191_MOESM11_ESM.pdf]
