# Supplementary material for: Age-related DNA methylation changes are tissue-specific with ELOVL2 promoter methylation as exception
Source: Epigenetics Chromatin. 2018 May 30;11:25. doi: 10.1186/s13072-018-0191-3 (PMC5975493; doi:10.1186/s13072-018-0191-3)
Supplement: Supplementary file 5 — Additional file 5: Figure S2. A Heatmap of slopes of aDMPs identified with only an effect size criterion. B Heatmap of slopes of aDMPs identified in equally sized datasets comprising randomly selected 96 individuals. Scale represents the change in DNA methylation in %/10 years. C Number of significant (P < 0.001) aDMPs in the other tissues in the equally-sized datasets. [file 13072_2018_191_MOESM5_ESM.pdf]

A

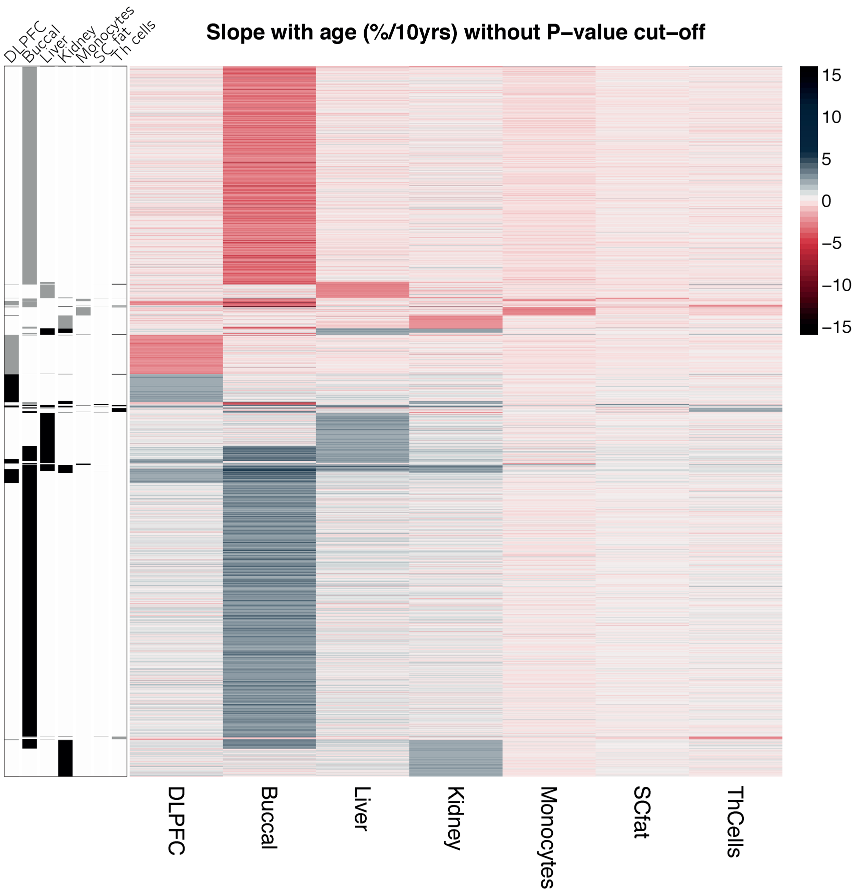


B

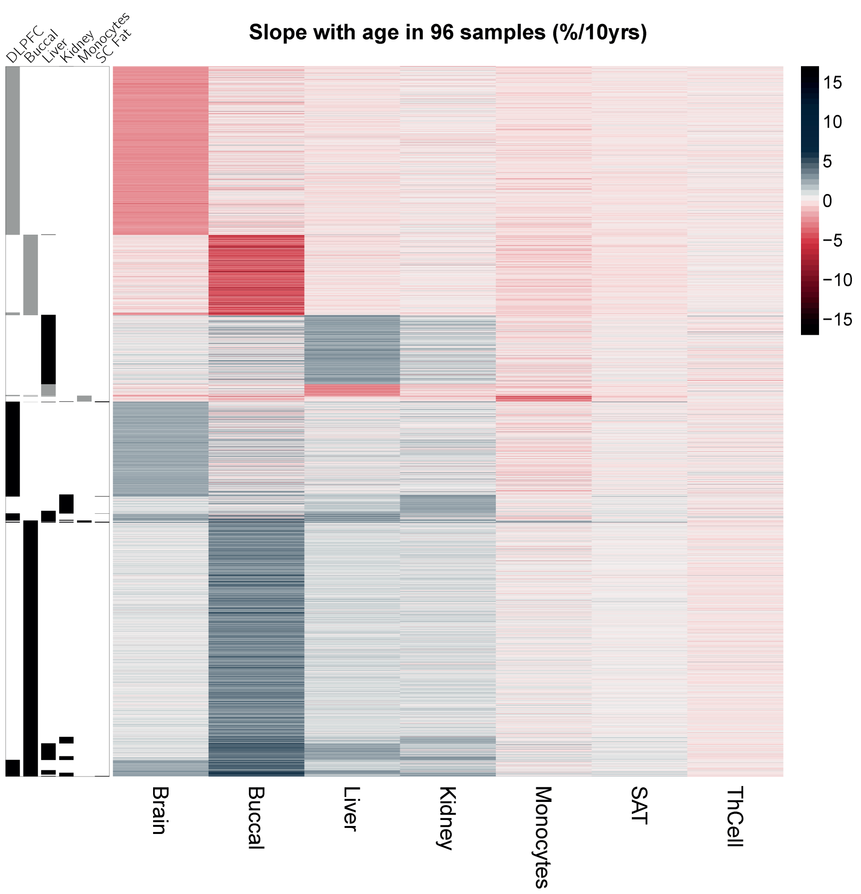


C

| | Brain | Buccal | Liver | Kidney | Monocytes | SC fat | Th cells |
|---|---|---|---|---|---|---|---|
| Brain (4622) | | 870 | 993 | 700 | 123 | 102 | 1 |
| Buccal (4857)) | 3309 | | 2568 | 1618 | 403 | 309 | 2 |
| Liver (2910) | 1703 | 964 | | 884 | 168 | 214 | 4 |
| Kidney (703) | 602 | 441 | 549 | | 87 | 195 | 1 |
| Monocytes (657) | 254 | 135 | 126 | 49 | | 37 | 0 |
| SC fat (59) | 54 | 38 | 44 | 38 | 21 | | 0 |
| ThCells (39) | 28 | 23 | 28 | 15 | 19 | 18 | |
