# Supplementary material for: Age-related DNA methylation changes are tissue-specific with ELOVL2 promoter methylation as exception
Source: Epigenetics Chromatin. 2018 May 30;11:25. doi: 10.1186/s13072-018-0191-3 (PMC5975493; doi:10.1186/s13072-018-0191-3)

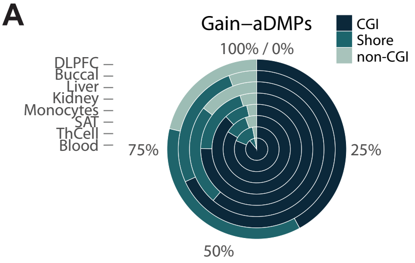

Tissue	CGI	Shore	n-CGI
Brain	1.6	1.2	0.5
Buccal	4.7	0.7	0.1
Liver	3.4	0.7	0.3
Kidney	6.5	0.5	0.1
Monocytes	15.6	0.2	0.1
SC Fat	10.9	0.3	0.1
Th cell	9.3	0.4	0.1
Blood	17.5	0.2	0.1

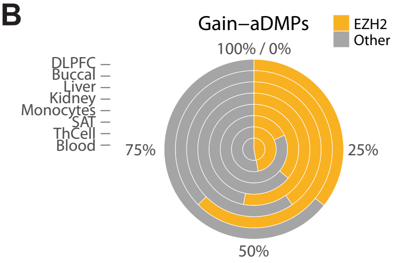

**Tissue**	**OR**
Brain	4.1
Buccal	12.7
Liver	5.0
Kidney	8.2
SC Fat	1.6
ThCell	6.4
Monocytes	4.1
Blood	6.5

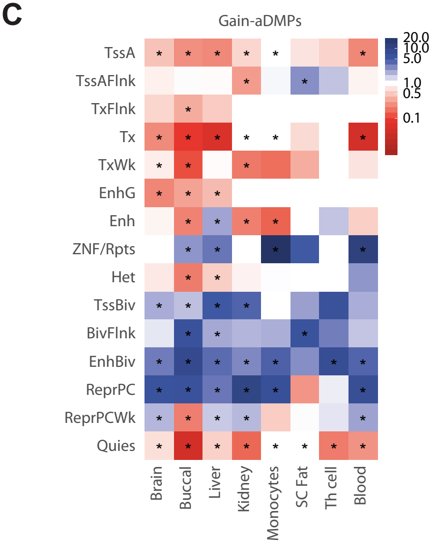

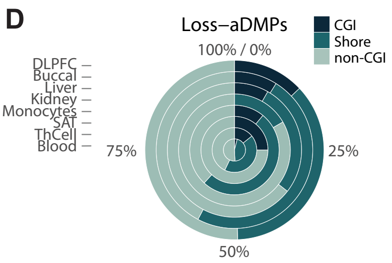

Tissue	CGI	Shore	n-CGI
Brain	0.3	1.2	1.9
Buccal	0.2	2.0	1.3
Liver	0.2	0.8	3.3
Kidney	-	0.4	9.1
Monocytes	0.3	2.1	1.1
SC Fat	0.7	-	5.5
Th cell	0.4	1.5	1.4
Blood	0.1	1.7	1.9

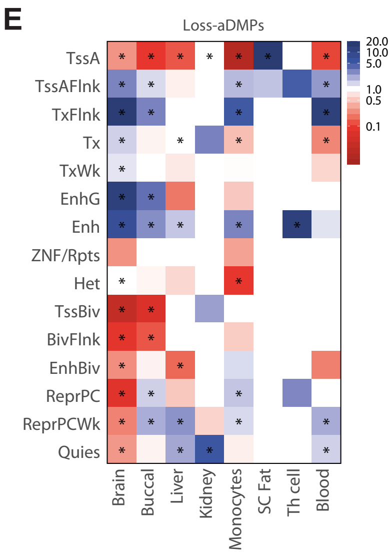

Supplement: Supplementary file 10 — Additional file 10: Figure S8. A Percentage (top) and odds ratios (bottom) of gain-aDMPs in CGIs, shores and non-CGIs. Blue enriched, red depleted, grey non-significant. B Percentage (top) and odds ratios (bottom) of aDMPs in EZH2 binding sites in the seven tissues plus whole blood (ChIP-seq, any cell type, ENCODE). Blue enriched, red depleted, grey non-significant. C Enrichment of gain-aDMPs in chromatin segmentations expressed in the seven tissues plus whole blood as an odds ratio, grey non-significant. D Percentage (top) and odds ratios (bottom) of loss-aDMPs in CGIs, shores and non-CGIs. Blue enriched, red depleted, grey non-significant. E Enrichment of loss-aDMPs in chromatin segmentations expressed in the seven tissues plus whole blood as an odds ratio, grey non-significant. Abbreviations: TssA, Active TSS; TssAFlnk, Flanking active TSS; TxFlnk, Transcr. at gene 5′ and 3′; Tx, Strong transcription; TxWk, Weak transcription; EnhG, Genic enhancers; Enh, Enhancers; ZNF/Rpts, ZNF genes + repeats; Het, Heterochromatin; TssBiv, Bivalent/Poised TSS; BivFlnk, Flanking bivalent TSS/Enh; EnhBiv, Bivalent enhancer; ReprPC, Repressed Polycomb; ReprPCWk, Weak repressed Polycomb, Quies, Quiescent/low. [file 13072_2018_191_MOESM10_ESM.pdf]
